# Understanding the Nature of Family Violence Against Women With Insecure Migration Status in Australia

**DOI:** 10.1177/10778012231199107

**Published:** 2023-09-14

**Authors:** Stefani Vasil

**Affiliations:** Monash Gender and Family Violence Prevention Centre, 2541 Monash University, Clayton, Victoria, Australia

**Keywords:** migrant women, domestic and family violence, migration policies, migration status, intersectionality

## Abstract

Feminist researchers have diversified understandings of family violence by examining how women's experiences are influenced by gender and its intersections with other social inequalities. This article seeks to contribute to intersectional and transnational feminist scholarship on violence that examines the influence of structural factors such as insecure migration status on the nature of women's lived experiences in Western industrialized countries. It reports on findings from a study with migrant women who experienced family violence in Victoria, Australia when their migration status was “insecure,” and examines similarities and differences in the forms and patterns of violence and abuse women described.

## Introduction

Feminist researchers have continued to diversify their understandings of family violence by examining how women's lived experiences are influenced by gender and its intersections with other structural inequalities related to race and ethnicity, class, sexuality, and citizenship (Abraham & Tastsoglou, 2016). Research on family violence among migrant women in Western industrialized countries including Australia, Canada, New Zealand, the United Kingdom (UK), and the United States (US), has more recently acknowledged the significance of “immigration” as a social location distinct from race or ethnicity (e.g., Erez et al., 2009; Pearce & Sokoloff, 2013; Sokoloff, 2008). Work of this kind has drawn attention to the ways that structural factors such as insecure migration status shape the nature and dynamics of women's lived experiences and their opportunities for help-seeking in different contexts.

Addressing the problem of family violence has continued to receive significant government attention in countries including Australia in recent years, and feminist researchers have highlighted the need to tailor policy approaches in ways that address the diversity and specificity of women's lived experiences (Abraham & Tastsoglou, 2016; Ghafournia & Easteal, 2018; Murdolo & Quiazon, 2015). While federal, state, and territory governments have established that family violence is prevalent in all Australian communities and have implemented different action plans for reform, there continue to be gaps in understanding of the nature of migrant women's experiences and how these are shaped by structural inequalities at a range of levels. This has resulted in what Murdolo and Quiazon (2015, p. 46) describe as a “limited theoretical and experiential knowledge base” from which to develop effective policy approaches to address and respond to violence, with little also known about the negative social consequences of mainstream policies, laws, and interventions for migrant women (Abraham & Tastsoglou, 2016; Walklate & Fitz-Gibbon, 2019).

This article seeks to contribute to understanding of the nature of family violence for migrant women who move to Australia on a range of temporary and provisional visas.^1^ Drawing on interviews with 18 victim-survivors with insecure migration status living in Victoria, Australia, I explore the similarities and differences in the forms and patterns of violence women described. I show that while the women interviewed moved for a range of reasons, held a variety of visas, and differed according to ethnicity and national origin, there were commonalities in the family violence they reported experiencing in Australia. I also show that the move to Australia intersected with family violence in specific ways and in doing so consider the influence of women's insecure migration status on the nature of their experiences. The article highlights that although there were similarities in women's accounts, the effects of insecure migration status on the lived experience of violence differed depending on women's individual circumstances and the resources that were available to them following the move (Bosniak, 2006). I conclude that a broader conceptualization of migration status can help to enhance understanding of the diversity and specificity of the family violence experience for different groups of migrant women. I suggest that this involves analyzing women's standing in a relational way by considering how they are positioned within intimate relationships, families, and ethnic, national, and transnational communities (Yuval-Davis, 2007) that afford them different rights and degrees of security over time and as they move. This analysis seeks to contribute to existing feminist scholarship that takes an intersectional and transnational approach and challenges essentialism by highlighting the diversity and complexity of the migration and family violence experience (e.g., Abraham & Tastsoglou, 2016; Sokoloff, 2008).

The next section of the article summarizes the literature on family violence for women with insecure migration status and focuses on studies that qualitatively examine the nature of their experiences in Western industrialized countries. The article then provides an overview of the chosen research design. Next, it discusses key findings that relate to women's lived experiences of violence, focusing on the different types they described and the ways these intersected over time. The article concludes with a discussion of the influence of migration status on the nature of migrant women's experiences in Australia. Here, I consider the value of an intersectional perspective, which recognizes the diversity of family violence, as well as the need to attend to the complexity of the experiences of migrant women who are disadvantaged in different ways by their structural location in society. The conclusion summarizes the key findings and implications for research and policy.

## Literature Review

Feminist research in Western industrialized countries has qualitatively examined how family violence manifests against women in the migration context.^2^ In their review of the literature, Menjívar and Salcido (2002, p. 900) point out that while “patriarchal ideologies” are expressed in different ways in local contexts, empirical examinations have sought to better understand the commonalities of migrant women's experiences of domestic violence “irrespective of context or culture.” Since this time, researchers have drawn attention to the ways that women's status as migrants intersects with gender and other structural inequalities to shape the nature of the family violence experience, including the forms and patterns of violence they are exposed to following the move (e.g., Anitha, 2011, 2019; Erez et al., 2009; Erez & Harper, 2018; Pearce & Sokoloff, 2013).

It is currently unclear whether migrant women experience more or less violence than victim-survivors from nonmigrant groups (Erez et al., 2009; Erez & Harper, 2018; Vaughan et al., 2015). Qualitative studies highlight that migrant women report experiencing many of the same forms of violence as other victim-survivors in the community, including violence that is physical, sexual, social, emotional, economic, technological, and related to reproduction (see e.g., Erez et al., 2009; Segrave, 2017; Vaughan et al., 2015). More recent attention to the dynamics of coercive control has furthered understanding of the patterns of violence and abuse against migrant women, including how perpetrators use migration systems “to threaten, coerce and control women” (Segrave, 2021, p. 26; see also Anitha, 2019; McIlwaine et al., 2019; Segrave et al., 2021; Singh & Sidhu, 2020). As part of this, feminist scholars have also contributed to the identification of new forms of violence. One example is transnational spouse abandonment, which is facilitated by the ways that gendered inequalities create conditions that enable men to abandon women in countries of origin and divorce them, leaving women in a precarious state by denying them access to residency and undermining their economic security (Anitha et al., 2018a 2018b; see also Segrave, 2021).

A growing number of studies in Australia and internationally has also focused on how issues regarding women's insecure migration status shape the nature of their family violence experiences (Anitha, 2010, 2011, 2019; Ghafournia, 2011; Gray et al., 2014; Mahapatra & Rai, 2019; Maher & Segrave, 2018; McIlwaine et al., 2019; Segrave, 2017, 2018, 2021; Vaughan et al., 2016; Voolma, 2018). Empirical research has, for example, examined the relationship between social isolation and sexual violence, which has been shown to manifest in distinct ways against women who are subject to visa sponsorship (e.g., Abraham, 1999, 2000; Anitha, 2011). Studies have also documented how sponsorship practices intersect with gender power dynamics and manifest as migration-related controlling behaviors, which include the refusal to sponsor women as well as threats regarding the withdrawal of sponsorship, deportation, and women's separation from their children (Segrave et al., 2021; see also McIlwaine et al., 2019; Segrave, 2017, 2018). Although fewer studies have documented the family violence experiences of migrant workers and international students, existing scholarship indicates that they report experiencing different forms of violence prior to, during migration, and following their arrival in host societies (e.g., domestic and sexual violence, human trafficking, state violence, as well as other forms of discrimination, including harassment and exploitation in public spaces and in the workforce; e.g., Cook Heffron, 2019; Forbes-Mewett & McCulloch, 2016; Robillard et al., 2018; Vaughan et al., 2016; Villegas, 2019). In their participatory research, Vaughan et al. (2016) also found that in addition to violence that was physical, sexual, emotional, and financial, women on temporary visas (e.g., partner, student, bridging, and work visas [*n* = 13]) reported that they were disadvantaged by the ways men used misinformation and intimidation regarding their citizenship or immigration status against them, which included threats related to the custody of children. Victim-survivors also described experiencing abuse by multiple family members, which extended to the family in countries of origin and was perpetrated using digital technologies (Vaughan et al., 2016; see also Henry et al., 2022; Segrave, 2017). It is owing to this confluence of factors that some researchers have suggested that insecure migration status can result in the intensification of the family violence experience and produce barriers that influence women's help-seeking and the resistance strategies they are able to rely on in their searches for safety (Anitha, 2011; Erez & Harper, 2018; Maher & Segrave, 2018).

While existing feminist scholarship has made significant contributions to knowledge about how family violence manifests against women with insecure status, studies have tended to draw from stakeholder or practitioner perspectives or are in the form of legal or policy analysis. Limited Australian research has so far been undertaken with victim-survivors to understand the nature of their lived experiences (exceptions include Vaughan et al., 2016). I seek to build on understanding in this article by drawing from women's direct accounts of violence and how these manifested in the context of migration.

## Method

### Research Aims and Terminology

The present study sought to contribute to understanding of the nature of family violence for migrant women with insecure status in Australia. “Migration status” has multiple meanings and is used in the present study to describe a legal category that refers to a way of being present in a particular jurisdiction or citizenship community (Bosniak, 2006). As others have demonstrated, the state confers upon individuals a diverse range of statuses that are associated with limits on citizenship, rights, protections, and access to services (e.g., Goldring et al., 2009). Changes to the structure of the Australian Migration Program in recent decades have seen the prioritization of skilled migrants for permanent settlement, further restrictions on the rights of family migrants, and an increased reliance on temporary migration (Hugo, 2014). This has contributed to the diversification of legal status categories (Robertson, 2019) and an increase in the proportion of migrants who are living and working in the Australian community with limited entitlements to permanent residency and settlement support (Tazreiter, 2019). While scholars such as Voolma (2018) have pointed out that insecure migration status is a legal term in the UK that refers to non-citizens who lack settlement rights, it is used in the present study not as an official term but to refer to a diverse range of impermanent statuses that are: *dependent or conditional* (e.g., partner and prospective marriage visa holders); *temporary* (e.g., work, student, and visitor visa holders); in *transition* (e.g., bridging visa holders); or *undocumented* (e.g., migrants with no valid visa). Also included in this definition are women—including permanent residents—who are *unaware of their migration status* (e.g., partners of permanent skilled migrants who believed their status was dependent; see for further discussion Vasil, 2023). These statuses determine the length of time individuals and their families are permitted to live in the community, the nature of their participation in the labor market, and their extent of access to public goods and services, such as social security payments, housing, education, healthcare, and legal, settlement, and other social supports (see for further discussion National Advocacy Group on Women on Temporary Visas Experiencing Violence, 2019).^3^

### Research Design

This article reports on findings from interviews with victim-survivors from a larger study that explored migrant women's experiences of family violence, help-seeking, and accessing formal support in Victoria, Australia (see Vasil, 2023). The first phase of the research involved interviews with 23 professional stakeholders and qualitative observation at sector events, seminars, and community meetings across Victoria. Interviews with migrant women were conducted in the second phase, and I report on the findings from this phase of the research in this article.

The chosen methodology was qualitative, and the research was also guided by the principles of feminist standpoint theory (e.g., Collins, 2000). Standpoint theorists view knowledge as socially situated and value women's experiences as credible sources of knowledge (Hesse-Biber, 2012). Standpoint theory gives authority to the claim that it is women's subordinated position vis-à-vis dominant groups that give them access to a “nuanced understanding of social reality” (Hesse-Biber, 2012, p. 11). I have drawn from the work of critical standpoint theorists who reject essentialism, recognize the complexity of social inequality, and consider how alternative sources of knowledge have the potential to challenge the status quo (e.g., Collins, 2000). By bringing migration status to the fore, I have sought to undertake an analysis of the family violence experiences of victim-survivors at “neglected points of intersection” in order to document “the differences and complexities of experience embodied in that location” (McCall, 2005, p. 1782) and build on understanding of the ways that violence manifests in the context of non-citizenship (Abraham, 2000).

### Participants

Semi-structured interviews were conducted with 18 women who expressed interest in participating in the project. All women were over the age of 18 years, living in Victoria, and had reported experiencing family violence in Australia when their migration status was “insecure.” Women differed in terms of their nationality, religion, and cultural heritage (see Table 1) and held a variety of visas (see Table 2). For all participants, English was not their first language, and most were between the ages of 20 and 38 at the time of the interview. Three women explained that prior to migrating to the country, they were living, working, and/or studying abroad, while 15 women indicated that they had migrated from their country of birth. All women had completed secondary school and the majority had also undertaken a university degree, with five women holding a postgraduate degree.

**Table 1.** Women's Nationalities.

Country of origin	No. of participants
India	4
Pakistan	3
Philippines	2
Sri Lanka	2
Thailand	2
Armenia	1
Bangladesh	1
Fiji	1
Malaysia	1
South Sudan	1

**Table 2.** Migration Status and Visa Class at Time of Arrival.

Migration status	No. of participants
Temporary	11
Student visa	5
Student visa (secondary applicant)	1
Tourist visa	5
Dependent	6
Partner visa (offshore)	4
Prospective marriage visa	2
Permanent	1
Skilled visa	1

In discussions about women's lives before arriving in Australia for the first time, most said that either they or their families were financially well off. A smaller number indicated that they had experienced financial hardship in their country of origin. Women's occupations were also diverse prior to arrival. Thirteen women were married, two were engaged, and one was partnered prior to migrating to Australia. Women who were in relationships before moving tended to be of the same ethnicity as their partners. Others were in cross-cultural relationships with immigrant men from Indonesia, Italy, and Greece, as well as Australian-born men with Anglo-Celtic heritage. Nine women had children, three of whom had their first child after migrating. At the time of the interviews, one woman was a naturalized citizen, nine women had secured permanent residency, and one was waiting on the grant of her permanent visa. Another two stated that their status was temporary, four were on bridging visas awaiting the outcome of a visa application, and one was holding a partner visa.

### Data Collection and Analysis

Formal ethics approval for this research was obtained from a university Human Ethics Committee and the interviews were conducted between October 2018 and April 2019. Victim-survivors were recruited in different ways, including through networks with professional stakeholders via dissemination of a project flyer with my contact details. I also sought permission to disseminate copies of the flyer via social media and in hard copy at targeted locations, such as libraries and community centers. Additionally, victim-survivors were recruited through word of mouth. A screening call with a list of eligibility questions was implemented and I provided verbal and written information in plain English so women could make an informed decision about participation. Interviews were conducted in a safe and private location and at a time of the participant's choosing, and strategies (such as data de-identification and the use of pseudonyms) were implemented to ensure confidentiality. A distress protocol was also implemented, and participants could elect to have a support person attend the interview with them and/or a female interpreter (from a different state if required).

Prior to the commencement of each interview, women's rights as participants were discussed and they were reminded that they did not have to answer a question if they did not wish to and could stop the interview at any time. Questions in interviews explored women's experiences of life prior to migrating and following the move to Australia, as well as their experiences of and responses to family violence. Interviews were transcribed verbatim and coded using NVivo software. Thematic analysis was used to analyze the data (Braun & Clarke, 2019). The next section of the article discusses findings relating to the nature of migrant women's lived experiences of family violence and uses direct quotes throughout to highlight their accounts.

## Findings: Family Violence Against Women With Insecure Migration Status

### Women's Initial Experiences of Life in Australia

The women interviewed in this study migrated for a range of reasons and under different circumstances. They also held a variety of visas and were at different life stages. Some explained that they had migrated to pursue further education while others said that they had come to the country for or as a result of marriage. Other participants stated that they moved for economic reasons with the intention of settling permanently.

Women also took on a variety of roles prior to the move, including as workers, students, wives, carers, heads of households, and mothers. While some women spoke positively about family life before migrating, several others described rigid gender expectations. When asked about their initial experiences of life in Australia, most women discussed the impact of the move on the dynamics of their intimate relationships. Many explained that their partner's behavior changed and escalated quickly in the weeks and months following their arrival in Australia. This was especially clear in the stories recounted by women who were married in the country of origin and remained there, awaiting the outcome of their visa applications, however, women in long-term relationships also described the ways that gender roles and responsibilities changed following migration.

Eight women explained that their experiences of family violence began prior to migrating. Of these women, half were in long-term relationships and half were recently married. All of these women said that their husbands exhibited controlling behaviors prior to the move, however, some also stated that they had experienced verbal and psychological abuse, as well as forms of economic control and exploitation, which was also perpetrated by more than one family member on their partner's side. Ten women said that they experienced family violence for the first time in Australia. This included women in new relationships who were recently engaged or married, as well as those who became partnered in Australia. Women who initially moved for the purposes of study and work and had become partnered in the community explained that they had fewer freedoms than they did previously. As I detail in the remainder of the article, all women reported experiencing different forms of violence, abuse, and coercive control in the weeks, months, and years following the move.

### Women's Migration Pathways and Experiences of Violence and Abuse

All victim-survivors interviewed described how their partners exercised control over their everyday lives in Australia. Danah, for example, arrived on a partner visa and explained that her husband exhibited controlling behaviors in the country of origin. She also described that his acts of control intensified after she settled in Melbourne: “When I came here in the first few months … that was not a good time because as soon as I arrived here my husband started controlling me a lot in every sense like socially, financially … it was like I was suffocated. I could not breathe.” Ananya migrated with her young child on a skilled visa and explained that her husband became increasingly controlling after she arrived, monitoring her everyday movements: “my passport, my email, my phone password, everything he control, he operates. He can access my phone … he put the password … he can access the emails … everything.” Some women who were partnered prior to migrating explained that they first experienced violence in the form of controlling and threatening behaviors after they arrived and were living with their partners in Australia. Gayathri, for example, held an offshore partner visa and expressed that she felt trapped by her husband's control tactics in the weeks following the move. She explained that he had not exhibited these behaviors in the lead-up to and after their marriage, which took place overseas, but that the relationship dynamics shifted after she arrived in Melbourne. Gayathri described the ways her husband sought to diminish her sense of autonomy by micromanaging her everyday life. A similar account was provided by women such as Cristina, who initially arrived in Australia on an international student visa and was in a long-distance relationship with an Anglo-Australian man prior to moving. She expressed that:

It felt like he was controlling my entire life, he drove me to Uni, everywhere I went. I was not allowed to work, and he constantly checked my phone, my laptop … and because I had no one else to depend on … I depended on him. I felt like I had no other choice, and I was also staying at his place, so … I felt like he had control over me, like he would just take away my phone or just lock me in the house or something.

Women also described how these acts of control intersected with their migration status; for all victim-survivors, migration status was a factor that contributed to and shaped the nature of their experiences and impacted them in different ways.

Victim-survivors discussed how perpetrators made threats regarding their migration status. These threats manifested differently depending on a victim-survivor's individual circumstances, including the specific visa they held, and whether they had children and family support. Women with dependent migration status (e.g., on partner or prospective marriage visas) experienced threats regarding the withdrawal of sponsorship, with perpetrators using their elevated status (e.g., as citizens or permanent residents) against them, making explicit threats to have women removed from the country. This had specific implications for mothers who were at risk of being separated from their children (see Segrave, 2018) or for women who were at risk of further violence if they were forced to return home (see Segrave, 2021).

Other women, including those on temporary visas, described how their partners made threats to return the family to their countries of origin, despite women's efforts to build a life for themselves in Australia. By withholding or restricting access to money, perpetrators were able to exercise significant control over women's migration trajectories, including the likelihood that they would be able to retain their status and continue living in the country. Maryam, for example, explained that her husband tried to sabotage her attempts to settle permanently by refusing to share his income when visas were due for renewal. He also made explicit threats to return the family back to Pakistan. While Maryam drew on different strategies to counter her husband's violence and was able to transition onto a permanent visa after several years, other victim-survivors explained that their migration status was directly affected by men's coercive and controlling behaviors.

Perpetrators who were sponsoring partners were able to capitalize on anti-immigrant sentiment, making verbal threats to report women to immigration authorities for various reasons even though their claims (e.g., that women had entered a relationship “fraudulently”) were unfounded. While most of these threats did not eventuate, three women on partner visas revealed that sponsorship was formally withdrawn and one woman on a temporary visa was removed as a secondary applicant along with her children without her knowledge. Jasveen, who was on a tourist visa, also revealed that her partner visa application was never formally lodged by her husband even though he and members of his family had made threats regarding the withdrawal of sponsorship as part of the exercise of control.

Women discussed the other ways that family violence impacted their migration pathways, including how male abusers arranged for them to travel to Australia on tourist visas instead of partner visas, as these are associated with fewer protections. Mary reflected on the ways that men use women's insecure migration status to manipulate them, expressing that: “they are using [us] because we are vulnerable because of that visa and they think that they can easily send you back, because you are just on that tourist visa.” Another woman, Fatima, also explained that she had been subjected to violence while living in Australia, was abandoned in the country of origin after she returned to visit family, and was subsequently prevented from re-entering Australia on her partner visa as her husband withdrew sponsorship. While many women explained that they came to find they were lied to, perpetrators capitalized on their isolation and lack of knowledge about Australian society and drew upon norms in a woman's country of origin (e.g., in relation to child custody and divorce) to manipulate them.

Women's accounts highlight that threats regarding their migration status were also enacted by family members, including parents- and other in-laws, as well as stepchildren. Ananya was controlled by her husband and his parents. She stated that: “when my husband shifted over here [my in-laws] just keep telling me, ‘If you do this, that, I will tell my son to not bring you over there’ … my mother-in-law always threaten me like this.” In addition to violence by multiple family members, women's accounts also reveal the transnational aspects of their experiences. Prisha, who held a tourist visa before being formally sponsored, explained that her husband's abuse continued by phone and Skype when she returned to India. She expressed that she felt trapped; even though she was physically distant, she was not able to escape the abuse. Other women who were in long-distance relationships described how they were controlled by their parents-in-law in countries of origin, and this was made worse by their husbands in Australia who were in constant contact by phone. Victim-survivors in Australia also explained how family members living overseas (from their husband's side) were able to monitor and control their behavior and make threats regarding women's deportation using digital technologies (e.g., by phone, via Skype, and other apps). These threats also intersected with misinformation, which was used to instill fear. In addition to concealing correspondence from immigration authorities, perpetrators also wrongly conveyed or withheld information about women's rights in Australia and their access to services (e.g., to healthcare or English language classes for partner migrants).

### Women's Heightened Social Isolation

Victim-survivors described how perpetrators used different tactics to increase their isolation following the move. Many women explained that they had few support networks to draw from and while some had connections in Australia, these tended to be from their husband's side of the family. Prisha expressed that she was dependent on her husband for social interaction, and this was made worse by his attempts to isolate her further from her family in India:

He put my phone in his [glass of] alcohol – the one phone I had. After one month when I stay on tourist visa for the first time, he buy a sim and recharge [the phone] … but he was like abusing and talking with me … like mental torture … and he took the glass and put it in there. … after that, long time, he did not give me the phone. Three, four time I talk with my family on his phone.

Like Prisha, Ananya, Leila, Cristina, Joana, and Waan also recounted the ways that male abusers restricted access to and monitored their use of technology, such as mobile phones, computers, and social media apps (e.g., Skype). This limited the ways that women on different visas were able to interact with social networks in Australia and overseas, heightened their dependence on perpetrators in some instances, and further entrenched their sense of isolation.

Women also explained that perpetrators prevented them from making new connections and friendships after they had moved. Mina—a prospective marriage visa holder—explained that she had cousins in Australia and was looking forward to seeing them after she arrived, however, after marrying her fiancé, she found that his behavior changed. He became increasingly controlling and prevented her from leaving the house or having any contact with family:

He would not let me. I do not know why. He said, ‘if you go there, then do not come back’ … I came here, I know no one. I want to go out, I want to communicate, it is impossible to all the time be with him. I am a doctor—I am used to communicating with people. I was working all my life, about 30 years and then I sit at home and do nothing. … Even if I want to go to city, once a week or once in two weeks, ‘why you go there? Museums are for tourists, not for you, why you go there? You want to meet your boyfriend?’ It was very hard.

Some women also explained that they were confined to the home in the weeks and months following the move. Leila, who arrived in Australia for the first time on an offshore partner visa, said that it was her husband's family who isolated her. Leila was deliberately distanced from her support system back home and her husband and parents-in-law also prevented her from establishing new connections and friendships. Leila explained that she was often locked inside the house by her mother-in-law and if she deviated from the family's rules (e.g., misplaced or lost something in the house) she would be verbally or physically assaulted (e.g., yelled at, put down, slapped).

Women on temporary visas also described how they were isolated following the move. Sahar arrived in Australia with her young child as a secondary applicant on her husband's student visa. She explained that her husband did not want her to migrate and while he traveled to and from the country of origin to see her after they were married, he would not add her to his visa application. Once she finally obtained her visa, Sahar found herself cut off from her husband and his social environment, which was compounded by the fact that he was restricting her social interactions in and outside the family home. She explained that for 3 years:

I did not know what is Australia, what Melbourne is, where I am. I was just bound in the house. I still remember those days. … I was in the house he did not allow us to go anywhere, so I can get to know what he is doing outside and what Melbourne is, and I can get knowledge that if I am in trouble or something happen to me where to go. He did not allow me to, and I did not have any friends. Nobody. I always wait for my parents to call … they will call me or when he is at home I always tell him can I talk to my sister, to my mother. Sometimes he tells me he did not have credit and sometimes he make a call and then I talk.

Sahar said that her social isolation diminished only once she started working. For other women, their experiences of working outside the home did not necessarily reduce their social isolation, with many explaining that they did not have opportunities to socialize or develop friendships. Cristina, Maryam, and Jayani—all international students—stated that they were working outside the home (e.g., as cleaners, in hospitality, and in door-to-door sales). Between work, study, and care responsibilities, women explained that there was limited time to establish meaningful social connections with colleagues and friends (Vasil, 2023). In fact, Maryam stressed that she was overworked and that this was compounded by her partner's economic abuse, which also contributed to her sense of isolation.

### Undermining Women's Security Through Economic Abuse

Women in the present study were most likely to state that they had limited say over how money was spent and many were denied access to the family's finances altogether. Tammy, who originally arrived on a student visa, transitioned onto a prospective marriage visa after she became partnered in Australia years later. She described a range of control tactics, which included her husband's micromanagement of the household finances. Tammy also explained that she had restricted access to money after she became pregnant and was forced to quit her job due to health reasons. This made it difficult for her and her young child to meet their basic needs:

I stay at home … now I am like a dog because no money. … I need to ask [for] his money … I hardly go anywhere, isolated because he work, and we are not eligible for healthcare card, concession card so I need to ask for a train ticket … I need money for the supermarket … he [did not want] to pay, but I need money to buy nappy and formula, milk and everything. I said, ‘can I have $200 a week for everything for me and baby?’ Sometime he gave to me, sometime he forgot. I ask him one day, he was not happy so he just [wave] the bank note like this and throw it on the floor. … he said I was burden … he said to me I am useless, because I cannot generate income, but I look after our baby.

Women who were working outside the home were also subjected to forms of economic control. Ananya explained that her husband's economic abuse worsened following the move to Australia: “he took all my salaries from me … he put in his account—my salary—it goes straight away.” She also described that: “if I not allow him to put in his account, he not allow me to work.” Ananya challenged her husband's economic abuse, however, these efforts were not always successful: “I yell one day, ‘give me some of the money so that I can do something.’ Then he said to me, ‘let us open your account.’ Then he put his—he put all the passwords and everything. So eventually when he opened my account, when every salary comes it is gone – I was just completely lost.” Ananya recounted that when she was applying for work in Melbourne, her husband attempted to sabotage an upcoming job interview and he also threatened to move the family interstate after she secured the job: “he was so angry he said, ‘you would not able to do this’ and he was yelling, shouting, throwing stuff.” Ananya's experience was exacerbated by her geographic isolation from her parents, who were her main source of support. In addition to experiences of economic control, women also described how male abusers and their relatives exploited them for personal and financial gain.

Victim-survivors described the ways that their labor was exploited prior to and following the move to Australia and that the beneficiaries included their husbands and any relatives who were living in the family home or were visiting from other countries. Women who had relocated for or as a result of marriage tended to say that they were confined to and forced to work inside the home, while students tended to be exploited for their productive labor in the workforce (Vasil, 2023). Jasveen was married in India but initially arrived on a tourist visa. She explained that her husband's abuse became worse as soon as she moved to Australia. Jasveen was responsible for all domestic responsibilities, as well as caring for her young stepchildren and her husband's relatives, and was rarely permitted to leave the house. Jasveen explained how her husband demeaned and undermined her, which included denying her the “status” of being his wife, which was important to her:

When I came here … this is the very worst time in my life … they expected me [to be] like I am like a chef, you know? Different meals, need to satisfy everyone. Need to clean, the washing, need to do everything, but he is not introduce me as a wife. Like in Centrelink, he show that he is single and that he has not married because he get the benefit from Centrelink as a single parent. When someone is coming over, he tell me – ‘you need to just disappear,’ because he do not want to show me as a wife. It is very painful. And he tell to the kids, ‘when we going [out], just tell [people] she is aunty.’

Fatima, who arrived on a partner visa, also said that her husband's economic abuse became evident in the weeks following her first trip to Australia. She explained that a dowry was negotiated prior to her marriage but that her husband and his family demanded more money after she migrated. Like Jasveen, Fatima was exploited by her husband and members of his family who benefitted from her confinement to the domestic sphere and the nature and extent of her domestic labor. In interviews, women made connections between their unequal status in the home and the ways they were expected, pressured, or forced to engage in domestic work. Jasveen said:

I do everything in the new house … everyone has their own room. When I need to put my clothes, there is no space. I just put on the shelf, some clothes of mine. So, every time when I am coming, my clothes are on the floor … every time. I do everything, I give everything, you know. So now, how can my clothes be on the floor every time? So, one time I see my older one, put my clothes on the floor so I [ask her] why. She tell me, ‘this is mine, this is mine.’ I am just using the corner you know—where I put my clothes? So, I call to mother in-law and what she tell me? ‘Oh, the kids is kids what you do, you just collect your clothes and the store room, where is your store room, just on one shelf, you put your clothes in there.’ I am his wife, I need to do everything like work, everything … but I have no right to do anything for me.

Women, including Jasveen, also described the impact of these acts, which deprived them of their daily needs and undermined their sense of belonging and status within the family.

Economic exploitation manifested in different ways against temporary migrants including students, with victim-survivors describing how they were forced to work outside the home to support themselves and their families while they were studying. This was often in more than one low-paid, casual job, and was made worse by a perpetrator's economic control, which meant that migrants such as Maryam were required to cover the costs of the household expenses as well as their university tuition, while men kept their income for themselves. Maryam expressed that she felt “used” by her husband. Not only did he refuse to share his earnings, but he also threatened to return the family back to Pakistan, which meant that Maryam—who wanted to settle permanently in Australia—was forced to work through the night to earn enough money to support her children and ensure she passed her course so that her visa conditions were met:

He would say, ‘okay we will go back,’ but no, he never [meant it]. [It] was to threaten me, to keep quiet, not to ask for anything … He used me like you can use a tissue, you know? … And the way I worked … you cannot imagine. I used to work like 20 h out of 24 h. No emotional support, no financial support, no psychological support, no friends and no community. No nothing.

Other women such as Mary who was on a prospective marriage visa explained that they left successful careers in their country of origin to move. As Mary was prevented from working, she was also unable to support her eldest child who was completing tertiary studies overseas. After a while, she was able to convince her husband to let her work with his relatives in a family-run business, which enabled her to generate some income, however, this too proved difficult as she suspected she was being underpaid and only earned a small amount per day. It was within these contexts of controlling, threatening, and coercive behavior, isolation from sources of support, and economic control, exploitation, and deprivation, that many women were also subjected to other forms of violence, including physical and sexual violence.

### Women's Experiences of Physical and Sexual Violence

Most women interviewed (*n* = 15) explained that they were physically assaulted at some point during the relationship. Many described how perpetrators hit and threw things at them while also verbally abusing them, using put-downs and other insults. Physical violence was most often perpetrated in the privacy of the family home. In some instances, women were also physically assaulted in public places, such as their place of work. Mina explained that her husband would beat her frequently and that she was often left with bruises. Other women, including Leila, explained that the physical abuse they experienced was made worse by the involvement of other family members, including parents-in-law. Leila's account also highlights the complexity of women's experiences, with physical violence often accompanied by other forms of violence and abuse (e.g., controlling behaviors, including those that were migration-related, as well as verbal abuse, technology-facilitated abuse, and multiperpetrator violence).

Some women who were subjected to physical violence described the impact it had on them as well as their families in their countries of origin. Riya initially migrated as a student. She experienced family violence for the first time in Australia after she married her husband—a permanent resident—who she met once she moved back to India after graduating. Riya described the impact of her husband's physical violence on herself and members of her family. She explained that her husband's behavior toward her changed after she reunited with him in Australia. She was not allowed to work, and he made efforts to extract money from her parents who were living in India. Riya explained that she was subjected to ongoing physical violence and while her husband was known to police, little was done to intervene or help her. She described how the violence became increasingly severe and frequent and that she feared for her life: “As usual, he just wants to beat me and never let me eat … that day, we were [at the dining table], he nearly finished the meal, then he lift the chair to bang it on my head. He desperately wants to kill me or get rid of me.” Riya explained that her husband: “just did not let me do [anything], every time just beat [me], and said ‘get money, get money, get money.’ Then, it went to the height of it when he had got my parents into the picture, saying that … he will tell my parents he will torture their daughter, until all the property is signed under his name.” Riya's account is one example of the ways that men's violence has direct implications for women's safety and security and how this can involve members of women's families in countries of origin who are geographically distant, making it difficult to intervene.

Some of the women who experienced physical violence also stated that they were subjected to sexual violence. Several women explained that they were forced, expected, or pressured to have sex with their partners. Most women stated that they were subjected to sexual violence after migrating to the country. Gayathri had lived separately from her husband for a period after they were married, and he returned to Australia for work. She expressed that she had been subjected to sexual violence for the first time after the move. She explained how she was expected to have sex with her husband, which she described as a way of controlling and exerting his dominance over her. Jasveen was the only woman to disclose sexual violence prior to migrating. She described the pressure she felt after she married her husband in India and recounted that: “I just close [my] eyes because I have not any options, you know? It is very horrible.” Jasveen said that she often felt unsafe around her husband whose violence intensified in Australia. Like other women, Riya's husband used sexual violence, as well as verbal abuse (e.g., shouting, using aggressive language, and “put-downs”) and physical violence (e.g., throwing objects, slapping, and shoving) to threaten, degrade, deprive, and instill fear in her. These threats were made possible by her legal dependence on him and her limited options in both Australia and her country of origin.

## Discussion: The Influence of Migration Status on the Nature of Women's Experiences of Family Violence in Australia

Women with insecure migration status who participated in the present study were a diverse group who migrated in different ways and had different aspirations. While some moved to better their prospects, others moved for adventure and love, leaving behind their careers and support systems to resettle with their partners. Their migration trajectories also differed and most made various transcontinental moves between Australia, countries of origin, and other countries where they had previously lived and worked. After arriving in Australia, women moved across visas and were confronted with the ways that the migration system constrained and enabled their mobility as well as aspects of their intimate lives. Their experiences reinforce the importance of attending to complexity in any analysis of migration and of recognizing the heterogeneity of women's lived experiences.

Scholars such as Constable (2006) have argued that is unhelpful to rely on unidimensional categorizations of women's mobility in research on gendered migration. Instead, a focus on differences in women's reasons for movement and the conditions that enable them to migrate can help challenge limiting stereotypes. This includes stereotypes that draw from a modernization narrative and are informed by the logic of a “poor woman” from the global South moving “from a poor or ‘backwards’ country to work for or marry a richer person in a more ‘modern’ country” (Constable, 2006, p. 3). As Constable (2006, p. 3) has argued, these accounts of women's movement do not consider the differences that exist among and between women and how they exercise their agency in different contexts, prior to, during, and following the move. Diversity feminists have also challenged monolithic characterizations of Third World women, with Mohanty (2003) examining the marginalizing effects of scholarship that not only essentializes the feminist subject, but that discusses the experiences of Third World women in a way that others them or categorizes them as victims.

Many of these tensions are evident in debates on family violence, with scholars challenging the tendency to rely on essentialist understandings of gender and culture. Narayan (2000) has argued that one response to gender essentialism in feminist theory has been to focus on the differences between women. What emerged in response was a simplistic view of culture that has worked to construct Third World women as “other” (Narayan, 1997). Kapur (2002) has also argued that applying a culturalist frame has led to the construction of a “victim subject” and that this has contributed to the view that gender subordination is something that is integral to “other” cultures. Family violence research that adopts an intersectional approach has sought to destabilize limiting discourses by engaging in analysis that reveals the impact of structural and systemic factors on the forms of violence women are exposed to following the move (e.g., Abraham, 2000; Crenshaw, 1991). As part of this, existing scholarship has sought to account for the ways that the state can operate as a “violent force” in migrant women's lives (Price, 2012, p. 3) via restrictive policies that limit their legal, social, and economic rights and in doing so, empower perpetrators (Segrave, 2018, 2021).

Findings from the present study suggest that women's insecure migration status played a role in shaping the nature of their lived experiences of family violence in Australia, including how they were impacted by coercive and controlling behaviors, social isolation, economic control, as well as the extent to which male perpetrators and other family members were able to control and limit women in their enactment of everyday life. This supports existing findings which highlight the ways that state policies can “exacerbat[e] structures of patriarchy within minority communities” (Anitha, 2011, p. 1260; see also Segrave, 2017, 2021; Voolma, 2018). In her research with South Asian marriage migrants in the UK, Anitha (2011, p. 1260) found that women's insecure immigration status played a role in “intensifying” the violence and abuse women were exposed to, and scholars such as Segrave (2017, 2018) have similarly argued that migration status provides additional “leverage” for control.

Findings from the present study also highlight that while migration status impacted women's lived experiences of violence in Australia, it did so in different ways. While women reported experiencing similar forms of violence following the move, their experiences differed depending on their individual circumstances, and the ways these were mediated by factors, such as visa class, employment status, language, care responsibilities, and the nature of women's social networks, including their position in the family. For example, international students described how they were exploited for their productive labor in the workforce, while others, including women on partner or prospective marriage visas, described how they were used for their domestic labor in the home (see also Vasil, 2023). As such, while migrant women experienced distinctive forms of violence and abuse, the diversity of their experiences also highlights that the effects of migration status are not always consistent and are structured by the interplay of other aspects of social location including gender as well as ethnicity and class (see Kapur & Zajicek, 2018).

Women in the present study also discussed that the frequency and severity of the violence worsened over time, which was compounded by the use of digital technologies and the involvement of multiple family members in the abuse (see Segrave, 2017; Vaughan et al., 2016). Most women described how they were impacted by the ways that perpetrators harnessed technology as part of the exercise of control and that this “amplified” the effects of the abuse they were experiencing (Harris, 2018; Henry et al., 2022). Women's accounts also highlight how they were disadvantaged by the ways that the violence traversed the national border with some—including those who were awaiting formal sponsorship—expressing that male partners and other family members continued to control them even when they were geographically distant (see Segrave, 2021). Their accounts highlight the transnational nature of family violence and the ways that they were simultaneously impacted by patriarchal arrangements in the home—which encompassed a variety of actors and occurred across different sites and scales (Mahler & Pessar, 2001; McIlwaine & Evans, 2020)—as well as the patriarchal authority of the state via restrictive migration and welfare policies, which were leveraged by perpetrators in the exercise of control (Segrave, 2018, 2021). This suggests that it was the ways that women were positioned within the family and different ethnic, national, and transnational communities (Yuval-Davis, 2007) that also contributed to and shaped the nature of their experiences, including the rights they were afforded and the resources they were able to draw on for support in Australia. These findings highlight the need to consider migrant women's standing in a relational way in research and policy, which involves examining the sets of relationships that characterize women's lives and intersect with state policies to amplify patriarchal control within and across national borders (Segrave, 2021). Moreover, by highlighting the *commonalities* and *specificities* of family violence for migrant women (Anitha, 2019), it is possible to see how their experiences are distinctive, but also how they share features with the lived experiences of victim-survivors from non-migrant backgrounds. This can be used to help counter essentialist understandings of gender and culture at the policy level and destabilize limiting discourses that deny migrant women their agency (Maher & Segrave, 2018; Murdolo & Quiazon, 2015).

## Conclusion

This research sought to contribute to a growing body of intersectional feminist scholarship that analyzes the nature of the family violence experience for migrant women in Western industrialized countries (Sokoloff, 2008). I found that women with insecure migration status in Australia experienced multiple forms of family violence which intersected in different ways following the move and occurred across transnational space. Women's accounts show that multiple and overlapping systems of oppression can work against victim-survivors who lack formal citizenship status and can contribute to a sense of being legally present though structurally invisible. Findings also suggest that a broader conceptualization of migration status, which focuses on women's social positionings and considers their standing in a relational way, can enhance existing understandings of the migration and family violence experience. These findings have important policy implications.

Findings from the present study suggest that understanding women's structural location in society is required in the design of effective policy responses that seek to address family violence at a variety of levels. Drawing from intersectionality in a policy context requires that policymakers bring the experiences of migrant women to the center so as to more accurately account for the experiences of all women, rather than viewing some women's experiences as representative of the majority and other women's experiences as an “add on” (Murdolo & Quiazon, 2015). The present study found that this involves attending to the ways that family violence is impacted by migration status, which can limit women's autonomy, heighten the social isolation and control they are exposed to, and reduce their options when a partner is violent (see Anitha, 2011; Jayasuriya-Illesinghe, 2018). It also means attending to the transnational character of family violence for women with insecure migration status as this can compound their experiences in intimate relationships (see McIlwaine & Evans, 2020; Segrave, 2021). Future reforms need to focus on elevating migrant women's status by ensuring all victim-survivors in the community are afforded the same rights in family violence situations so as to prevent perpetrators from using women's status against them and to enable women to access the support they need before the violence reaches crisis point (Jelinic, 2019). Findings also highlight the need to move away from viewing difference through a culturalist lens, as this can overlook the ways that social structures position people unequally in systems of power (Young, 2000). Cultural stereotypes that rely on essentialism tend to invoke a modernization narrative, which influences how migrant women are represented in policy and the ways that resources are allocated to them (see Jayasuriya-Illesinghe, 2018). This can contribute to their exclusion from mainstream responses and impact the accessibility of different forms of family violence support. Viewing experiences of violence through this lens also fails to acknowledge the complexity of the lived experiences of migrant women who are disadvantaged in different ways by their structural location in society.

## Notes

1. My focus in this article is on the experiences of women who arrived in the country under the planned migration program (e.g., family migrants and a smaller number of skilled migrants who are granted provisional visas rather than PR on arrival), as well as the temporary residence program (e.g., migrants on student, work, and visitor visas). I took the view that migrants who arrive for humanitarian reasons and are granted temporary protection or bridging visas are faced with specific issues in Australia that distinguish their experiences from migrants who arrive under voluntary schemes (see Vasil, 2023). As such, their experiences are not reflected in this article.

2. I adopt a broad definition of family violence, which includes a diverse range of behaviors that are perpetrated by family members (including but not limited to intimate partners) and are physically, sexually, emotionally, psychologically, and economically violent, abusive, threatening, coercive, or controlling (*Family Violence Protection Act 2008* [Vic], s. 5).

3. While in Victoria, all victim-survivors are entitled to seek help from police or a frontline service, migration policies and restrictions on social welfare reduce the type and degree of support that migrant women can receive in practice. Migrant women have differential access to a range of social entitlements and services, such as public healthcare, as well as housing, legal and settlement support, and financial support (e.g., income supplements, single parent payments, and crisis support payments), which also impacts the likelihood that they will be able to be housed in a refuge. It is because of the conditions attached to women's visas that frontline family violence services are restricted in their ability to support them. This is exacerbated in instances where women have no access to income or financial assistance (VRCFV, 2016). While women on partner visas are eligible for specific protections in migration law (known as the “Family Violence Provisions”) and may be able to apply for an exemption to access the severe financial hardship payment known as Special Benefit, women who are not on this pathway have limited options (see Gray et al., 2014). In fact, women and their children who have no legal right to remain in the country may be forced to leave Australia if their circumstances change and their relationship breaks down.
